# Factors Associated With Positive Aging and Happiness of the Older People in Hong Kong

**DOI:** 10.1093/geroni/igaa057.378

**Published:** 2020-12-16

**Authors:** Daniel W L Lai, Vincent Lee, Elsie Yan

**Affiliations:** 1 The Hong Kong Polytechnic University, Kowloon, China; 2 The Hong Kong Polytechnic University, Hong Kong, China

## Abstract

Happiness is essential to one’s well-being and impact on every aspect of our lives. Happier people are living longer, they are healthier. Happier people are more likely to be physically active and enjoy better sleep habits and practices. While few existing research studies had examined the determinants of happiness of older people, especially in Chinese society. Understanding happiness in the context of social unrest and political instability is thus limited. This study tested the correlates of happiness at interpersonal, psychological and environmental levels at a time when there were extended scale of violence, destructions, and clashes in the community between police and protesters in late 2019 during the anti-extradition campaign. In social unrest, older people, due to their function and mobility, could be emotionally and physically vulnerable. A total of 1,209 older persons aged 55 and above from Hong Kong answered the questionnaire by stratified random sampling. Our findings show that their overall resilience was strongly and positively associated with levels of happiness. Due to the recent political instability Hong Kong, their satisfaction toward social and political situation of Hong Kong also correlated positively to levels of happiness. We suggest that future interventions and policy initiatives should put extra emotional and tangible support to older adults, particularly during social unrests and unstable political conditions, in addition to strategies for the enhancement of resilience and mental capital.

